# Bilateral internal carotid artery occlusion following embolization of the right internal carotid artery in a nasopharyngeal carcinoma with massive bleeding: a case report

**DOI:** 10.1016/j.bjorl.2026.101867

**Published:** 2026-07-24

**Authors:** Yu Wu, Qiang Luo, Qing He

**Affiliations:** a Chengdu Xinjin District Hospital of Traditional Chinese Medicine, Department of Oncology, Xinjin District, Chengdu, Sichuan Province, China; b West China Hospital of Sichuan University, Department of Head And Neck Oncology, Chengdu, Sichuan Province, China

## Introduction

Radiation-induced nasopharyngeal hemorrhage is a severe complication of nasopharyngeal carcinoma (NPC) treated with radiotherapy. This complication poses a substantial threat to patients' lives. A previous study reported that the mortality rate could be high up to 76% when NPC encountered internal carotid artery (ICA) rupture.^1^ With advancements in vascular interventions, the management of ICA rupture and hemorrhage has evolved, primarily utilizing endovascular techniques such as parent artery embolization or stent-assisted revascularization. However, outcomes for NPC patients with profuse epistaxis remain poor. Active prevention is superior to passive treatment; nevertheless, few investigations explore the feasibility of the early detection of vascular injuries and the prevention of bleeding.

## Case description

A 49-year-old woman was diagnosed with NPC (T4N1M0) in November 2021. Then, she underwent a concurrent chemoradiotherapy. Following treatment, she complained of mild headache, which gradually worsened despite symptomatic management. On April 11, 2023, the patient experienced spontaneous nasal bleeding (100 mL). Three days later, she had a recurrent episode of bleeding (400 mL) and was referred to our hospital. Upon presentation, she developed massive nasal bleeding (800 mL) and lost consciousness. Emergency angiography showed a dilated left vertebral artery and well-developed right cerebral arteries via the posterior communicating artery (Fig. 1A). Pseudoaneurysm was identified in the C3 segment of the right ICA (Fig. 1B). Coil embolization was performed urgently, resulting in resolution of the aneurysm (Fig. 1C) and complete cessation of bleeding. She was subsequently transferred to the Intensive Care Unit (ICU), where she received norepinephrine and metaraminol for hemodynamic support alongside other symptomatic and supportive interventions.

**Fig. 1:**
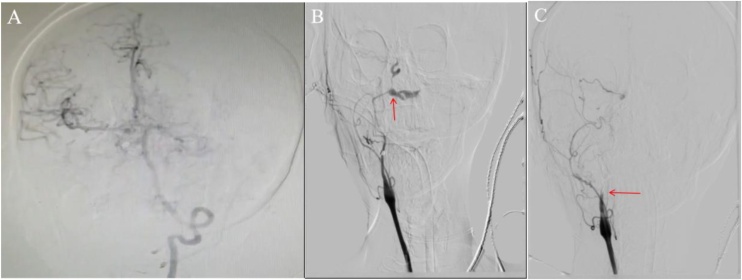
Intraoperative fluoroscopic imagings: (A) The left vertebral artery is thickened and tortuous. The right middle cerebral artery and anterior cerebral artery are well displayed through posterior communicating artery (red arrow). (B) Both the right internal and external carotid arteries were significantly stenosed with a pseudoaneurysm noted in the C3 segment of the right ICA (red arrow). (C) The pseudoaneurysm was embolized using spring coils and complete resolution was observed after embolization (red arrow).

Twenty-four hours later, the patient regained consciousness and was successfully extubated. However, computed tomography angiography revealed cerebral infarction in the right parietal lobe and left frontoparietal lobes (Fig. 2A), as well as bilateral ICA occlusion starting from the C1 segment (Fig. 2B).

**Fig. 2:**
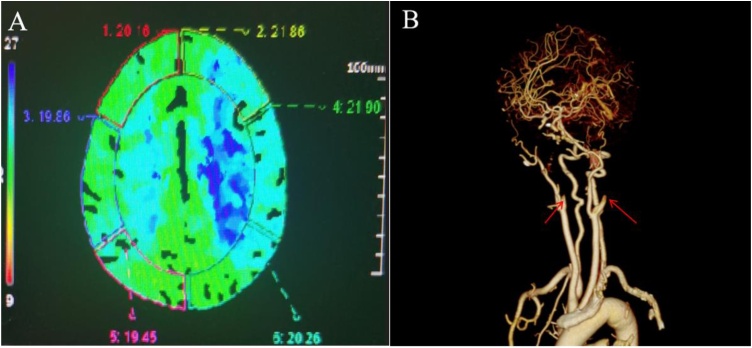
Post-operative imagings: (A) CT scan revealed cerebral infarction in the right parietal and left frontoparietal lobes. (B) Head and neck CTA showed bilateral ICA occlusion involving the C1-4 segments (red arrow).

Following multidisciplinary consultation, supportive treatment was initiated. Thirteen days later, the patient abruptly developed unconsciousness and hyperthermia (41 °C) without any inducements. After a comprehensive discussion of the patient’s prognosis and available treatment options, her family opted to withdraw treatment and pursue discharge. The patient succumbed to her condition 8 -hs later.

## Discussion

Over the past decade, the incidence and mortality of NPC have declined markedly, with radiotherapy playing a pivotal role in achieving this clinical outcome. However, radiotherapy is also associated with severe adverse events, including nasopharyngeal necrosis, nasal fetor and epistaxis. When necrosis invades ICA, the risk of mortality increases significantly.^2^ The overall incidence of severe epistaxis following radiotherapy for NPC has been reported to be approximately 2.7%, with 1% of cases attributed to ICA rupture.^3^ Additionally, radiotherapy elevates the risk of ICA rupture in NPC patients by nearly tenfold.^4^

Recently, interventional therapy has advanced considerably and emerged as a promising strategy for carotid artery rupture. Fluoroscopic endovascular treatment, in particular, has gained widespread acceptance due to its distinct advantages. However, outcomes for NPC patients with severe epistaxis remain poor. Zhao et al. reported that endovascular interventions for such cases are associated with a perioperative mortality rate of 11%, a neurological complication rate of 3% and a 39% survival rate.^5^ In emergency settings, we often encounter severe vasospasm and stenosis of the affected vessels with inadequate collateral circulation, rendering both embolization and stent deployment ineffective. This presents clinicians with a clinical dilemma: balancing the risk of fatal hemorrhage against that of cerebral infarction. Recognizing this challenge and initiating appropriate management is essential.

To our knowledge, this is the first report of bilateral ICA occlusion following unilateral ICA embolization in an NPC patient. To gain a deeper understanding of this clinical phenomenon, we conducted a comprehensive review of her imaging data from routine post-radiotherapy follow-up. Bilateral ICA C3 segment stenosis was detected at 1 month post-radiotherapy (8-months prior to the hemorrhagic event), whereas pre-radiotherapy vessels were normal in diameter (Fig. 3A‒B). Urgent angiography confirmed severe stenosis of the left ICA (Fig. 3C); accordingly, hemodynamic support such as norepinephrine and metaraminol administration may increase the risk of in-vessel thrombosis. This may explain the development of bilateral ICA occlusion subsequent to right ICA embolization. Given the 8-month interval between detection of ICA changes and fatal bleeding, earlier interventions (implementation of carotid compression test to achieve more adequate collateral circulation, or embolization/ stent deployment to protect and reconstruct infiltrated vessels) would likely yield safer and more effective outcomes compared with emergency endovascular intervention.

**Fig. 3:**
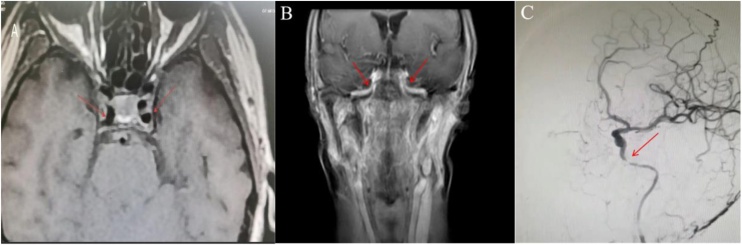
MRI findings before and after radiotherapy, and Intraoperative fluoroscopic imaging’s: (A) Pre-radiotherapy (April 2022): The C3 segment of both ICAs appeared normal, with a regular diameter. (B) One month after radiotherapy (August 2022): Bilateral stenosis of the ICA C3 segment was observed. (C) April 2023: Significant stenosis of the left ICA C3 segment was noted.

## Conclusion

Early detection enables early management. Upon detection of vascular lesions, active intervention can be implemented alongside more frequent follow-up, even in the absence of hemorrhage. Further research is required to validate the efficacy of early prophylactic interventions.

## ORCID ID

Qing He: 0000-0003-3452-856X

Yu Wu: 0000-0002-1329-2917

Qiang Luo: 0009-0001-8384-1475

## Authors’ contributions

Writing-original draft: Yu Wu. Writing-review & editing: Qing He. Data curation and investigation: Qiang Luo.

## Ethics approval and consent to participate

It is a retrospective research, so ethics approval is not needed. Written informed consent for publication was obtained from the patient’s family.

## Funding

None.

## Data availability statement

Data sharing is not applicable to this article as no data were created or analyzed in this study.

## Declaration of competing interest

The authors declare no conflicts of interest.

## References

[1] Xu X., Ong Y.K., Loh W.S., Anil G., Yap Q.V., Loh K.S. (2021). Clinical predictors of internal carotid artery blowout in patients with radiated nasopharyngeal carcinoma. Head Neck..

[2] Wu W.B., Zhang X.B., Liu Y.P. (2023). Stent pretreatment for internal carotid artery exposed to necrotic lesions in nasopharyngeal carcinoma. Rhinology..

[3] Li C., Lu J., Luo Y., Feng D. (2023). Case report: endovascular intervention of internal carotid artery pseudoaneurysm secondary to nasopharyngeal carcinoma radiotherapy. Front Surg..

[4] Xu X., Gopinathan A., Ong Y.K., Thye Thong M.K., Loh W.S., Loh K.S. (2023). Deconstructive versus reconstructive approach to internal carotid artery blowout in radiated nasopharyngeal carcinoma: a tertiary center experience and systematic review. Head Neck..

[5] Zhao Z., Huang L., Chen J. (2021). Comprehensive treatment strategy for internal carotid artery blowout syndrome caused by nasopharyngeal carcinoma. Otolaryngol Head Neck Surg.

